# Molecular Determinants of Target Cell Recognition by Human γδ T Cells

**DOI:** 10.3389/fimmu.2018.00929

**Published:** 2018-04-27

**Authors:** André E. Simões, Biagio Di Lorenzo, Bruno Silva-Santos

**Affiliations:** ^1^Faculdade de Medicina, Instituto de Medicina Molecular, Universidade de Lisboa, Lisbon, Portugal; ^2^Instituto Superior Técnico, Universidade de Lisboa, Lisbon, Portugal

**Keywords:** gamma-delta T cell, T cell receptor, NK cell receptor, NKG2D, tumor immunology

## Abstract

The unique capabilities of gamma-delta (γδ) T cells to recognize cells under stressed conditions, particularly infected or transformed cells, and killing them or regulating the immune response against them, paved the way to the development of promising therapeutic strategies for cancer and infectious diseases. From a mechanistic standpoint, numerous studies have unveiled a remarkable flexibility of γδ T cells in employing their T cell receptor and/or NK cell receptors for target cell recognition, even if the relevant ligands often remain uncertain. Here, we review the accumulated knowledge on the diverse mechanisms of target cell recognition by γδ T cells, focusing on human γδ T cells, to provide an integrated perspective of their therapeutic potential in cancer and infectious diseases.

## Introduction

More than three decades after the discovery of gamma-delta (γδ) T cells (1), the research community is still missing a compelling picture about their mechanisms of activation and target cell recognition. Despite the relatively small abundance of γδ T cells in the human blood, it is clear that this lymphocyte population plays an important role at the interface between the innate and the adaptive immune systems. These cells share T cell receptor (TCR) rearrangements and memory functions (2) with their αβ T cell counterparts, but differ in their response kinetics and mechanisms of target cell recognition. Thus, γδ T cell activation is typically independent of antigen presentation by major histocompatibility complex (MHC) molecules. Furthermore, γδ T cells bear a plethora of NK cell receptors (NKRs) on their surface, which allow for very fast responses against infected or transformed cells (3), thus contributing to a first line of defense that precedes antigen-specific αβ T-cell responses (4).

Unlike αβ T cells, there is little evidence of thymic negative selection of self-reactive γδ T cells. Vγ9Vδ2 T cells, which constitute the major (60–95%) γδ T cell subtype in humans, seemingly expand in the periphery in response to microbial or stress-induced phosphorylated antigens (2) while displaying preferential Vγ9-JP TCR rearrangements (5). Other human γδ T cell subsets, namely Vδ1^+^ and Vδ3^+^ T cells that are highly reactive to cytomegalovirus (CMV) infection (6), display TCR repertoires biased toward sequences recognizing CMV-infected cells (7). But while Vγ9Vδ2 TCR recognition has been well characterized and discussed (5, 8), it remains less clear how other γδ T cell subsets are activated to participate in lymphoid stress surveillance (9).

The purpose of this review is to discuss the current knowledge on target cell recognition by human γδ T cells (Table 1), emphasizing the role of the TCR as well as NKRs and their ligands, in the context of cancer and infectious diseases.

**Table 1:** Tumor- or infected cell-associated ligands recognized by gamma-delta (γδ) T cells.

Ligand	Receptor	γδ subset	Infection/cancer	Reference
CD1 proteins + endogenous or exogenous lipids	T cell receptor (TCR)	Duodenal	Infection	(10, 11)
BTN3A1 + phosphoantigens	TCR	Vγ9Vδ2	Infection	(5)
Endothelial protein C receptor	TCR	Vγ4Vδ5	Both	(12)
Annexin A2	TCR	Vγ8Vδ3	Both	(13)
Heat shock protein 60	TCR		Both	(14–17)
F1-ATPase	TCR		Cancer	(18)
SEA and SEE	TCR		Infection	(19)
OXYS	TCR		Infection	(20)
DXS2	TCR		Infection	(21)
Glycoprotein I	TCR		Infection	(21)
MSH2	TCR		Both	(14, 22)
	NKG2D			(22)
HLA-E	NKG2C		Infection	(23)
HA	Sialic acid receptor		Infection	(24)
CD48	2B4		Cancer	(25–27)
MICA/MICB	TCR	Vδ1	Both	(28–30)
	NKG2D			(29–32)
MICA	NKG2D	Vγ9Vδ2	Cancer	(33)
UL16 binding protein (ULBP)1	NKG2D	Vγ9Vδ2	Cancer	(34)
ULBP2	NKG2D	Vδ1	Cancer	(35, 36)
ULBP3	NKG2D	Vδ1	Cancer	(35–37)
ULBP4	TCR and NKG2D	Vδ2	Cancer	(38)
?	NKp30	Vδ1	Both	(39, 40)
PVR/Nectin-2	DNAX accessory molecule 1	Vγ9Vδ2	Cancer	(41)

*“?” means undescribed/unknown in the referenced studies*.

## Tumor Cell Recognition

Early research on the molecular mechanisms of γδ T cell recognition in the 1990s led to the realization of its unusual independence of peptide processing and MHC-restricted presentation, in marked contrast with αβ T lymphocytes (42–44). One of the first lines of evidence came from non-peptidic prenyl pyrophosphates [“phosphoantigens” (PAg)] recognized by Vγ9Vδ2 TCRs (45, 46). Initially, bacteria and parasites were shown to produce strong PAg agonists for Vγ9Vδ2 TCRs (47), but later it became clear that these could also be activated by weaker agonists, such as isopentenyl pyrophosphate (IPP) and dimethylallyl pyrophosphate, that are natural intermediates of the mevalonate pathway of isoprenoid and steroid synthesis in eukaryotic cells (48). Importantly, the dysregulation of the mevalonate pathway in some tumor cells allows for the accumulation of these (weaker) PAgs, thus promoting Vγ9Vδ2 TCR-mediated recognition (49). Furthermore, treatment with zoledronate or pamidronate (which are approved drugs) was shown to be very effective at inducing the accumulation of intracellular PAgs like IPP, and thus potentiate TCR-dependent Vγ9Vδ2 T cell cytotoxicity against tumor cell targets, including cancer stem cells (50).

A key recent breakthrough was the discovery of butyrophilin-related proteins, especially BTN3A1, as major molecular determinants of Vγ9Vδ2TCR-mediated recognition of PAgs, even if the underlying mechanism has gathered some controversy. A model supporting extracellular PAg presentation to the Vγ9Vδ2 T cell (in a MHC-like manner) was first proposed, with biophysical and structural data in support (51). However, following reports demonstrated that PAgs interact directly with the intracellular B30.2 domain of BTN3A1 through a positively charged surface pocket; and that charge reversal of pocket residues abrogates PAg binding and Vγ9Vδ2 T cell activation, with no detectable association with the extracellular domain of BTN3A1 (13, 52, 53). More recently, it has been shown that changes in the juxtamembrane domain of BTN3A1, which is located close to the start of the B30.2 domain, induced marked alterations in Vγ9Vδ2 T cell reactivity, thus highlighting the importance of the intracellular domain for the correct Vγ9Vδ2 T cell function and activation (54). Because of its location between the intracellular and the extracellular domains, the B30.2 domain seems critical in translating the pAg-induced conformational change of BTN3A1 from the inside to the outside of the target cells (55, 56).

Besides sensing PAgs, γδ T cells seemingly recognize transformed cells through proteins that are expressed at the cell surface in a stress-induced manner. Some examples are typically endogenous proteins, such heat shock protein 60 (14–17) or FI-ATPase (18), that can be ectopically expressed on the cell membrane upon transformation and recognized by Vγ9Vδ2 TCRs to promote tumor cell lysis. More recently, endothelial protein C receptor (EPCR), which acts on the coagulation cascade, was shown to be exposed on the cell surface during transformation and recognized by a non-Vδ2 (Vγ4Vδ5) TCR (12). Similarly, Annexin A2, expressed on tumor cells in response to increasing quantities of reactive oxygen species, engaged directly with a Vγ8Vδ3 TCR (13). The identification of these rather different ligands highlights the complexity of tumor cell recognition *via* γδ TCRs. This notwithstanding, it is clear that γδ T cells also rely on “NK-like” mechanisms for tumor cell recognition, using receptors such as 2B4 and NKG2D, originally thought to be specific to NK cells.

The first indication of an NK-like recognition mechanism was unveiled upon the ability of stimulated murine γδ T cells to recognize CD48 (25, 26), a well-known 2B4 ligand, suggested to work as an accessory molecule that strengthens effector–target interactions (27). Surprisingly, only the 2B4^+^ γδ T cells were able to develop non-MHC-restricted cytotoxicity against lymphoma cells (57, 58). Although 2B4 is also expressed on activated human γδ T cells, its relevance is still unclear as 2B4 engagement failed to promote proliferation or cytokine production (59).

Much more consensual is the role of NKG2D, which is widely expressed not only in NK cells but also in most γδ and some αβ T cells (31, 60, 61). In human γδ T cells, both Vδ1^+^ and Vδ2^+^ subsets, NKG2D was shown to be responsible for recognition of tumor cells expressing MHC class I chain-related (MIC) A/B (28, 29, 31–33, 62) or UL16 binding protein (ULBP) 1/2/3/4 (34–38, 50, 63) ligands. In fact, human carcinoma samples from lung, breast, kidney, ovary, and prostate cancers expressing MICA or MICB presented higher levels of infiltrating Vδ1^+^ T cells, which in turn were able to target and kill autologous and heterologous tumor cells (25, 59). Our group’s work revealed that ULBP1 was particularly important for leukemia and lymphoma cell recognition by PAg-activated Vγ9Vδ2 T cells (34). Notwithstanding, one should note the relevance of a synergistic TCR engagement for an efficient cytotoxic response (37, 38). In fact, some works suggested that MIC or ULBP recognition by γδ T cells is not only restricted to NKG2D but also involves the γδ TCR (26, 31). A similar recognition pattern was also observed against human MutS homolog 2 (hMSH2) ectopically expressed in epithelial tumor cell lines. Both TCRγδ and NKG2D were able to interact with hMSH2 and contribute to Vδ2^+^ γδ T cell-mediated cytotoxicity and interferon γ (IFN-γ) production (14, 22).

Besides 2B4 and NKG2D, DNAX accessory molecule 1 (DNAM-1) was also shown to be widely expressed in Vδ1^+^, Vδ2^+^, and Vδ1^−^Vδ2^−^ γδ T cell subsets (64); and masking DNAM-1 on γδ T cells significantly inhibited tumor cell killing (64, 65). DNAM-1-dependent γδ T cell recognition was reported for hepatocellular carcinoma (41), acute (65) and chronic (64) myeloid leukemia, and multiple myeloma (66) cell lines. More specifically, Vγ9Vδ2 T cells were shown to use DNAM-1 to interact with Nectin-2 and PVR that are widely expressed in the tumor cell targets (41, 65). Curiously, PVR engagement potentiated γδ T cell cytotoxicity, whereas Nectin-2 blocking did not affect it (41). Tumor targets that expressed both DNAM-1 and NKG2D ligands were able to engage both receptors on γδ T cells, having a synergistic effect on their cytolytic activity (41, 64, 66). Moreover, therapeutic strategies that enhanced the expression of NKG2D or DNAM-1 ligands, such as MICA/B and ULBP1/2, or Nectin-2 and PVR, respectively, potentiated γδ T cell recognition of colon cancer, glioblastoma, multiple myeloma, and lymphoma cells (67–70).

From a therapeutic perspective, γδ T cell recognition of tumor cells may also rely on the induced expression of natural cytotoxicity receptors (NCRs) that recognize a distinct set of tumor-associated ligands, such as B7-H6 or BAT3 (71). Thus, our group has shown that NKp30 and NKp44 can be reproducibly induced *in vitro* in Vδ1^+^ (but not Vδ2^+^) γδ T cells (39). A very mild expression of NKp44 on expanded γδ T cells had been reported before (72); and shown to contribute γδ T cell cytotoxicity against myeloma cells (61). In our studies, we observed not only a robust expression of NKp44 but also NKp30, in Vδ1^+^ T cells activated with TCR agonists and IL-15 (or IL-2); and both receptors enhanced γδ T cytotoxicity against tumor target cells (39, 73). Among the various known ligands for NCRs, it is still unclear which are most relevant for NCR^+^ Vδ1^+^ T cell recognition of tumor cells. While the NKp30 ligand, B7-H6, is an obvious candidate (74), a very recent report identified an unanticipated ligand for NKp44 in the form of platelet-derived growth factor (PDGF)-DD (75), known for its capacity to promote of tumor cell proliferation, epithelial–mesenchymal transition, and angiogenesis. PDGF-DD ligation to NKp44 enhanced IFN-γ and TNF-α secretion (by NK cells), which in turn induced tumor cell growth arrest (75). Additional investigation will be needed to elucidate the relative importance of NCR, NKG2D, DNAM-1, or TCR ligands in tumor cell recognition by γδ T cells, aiming to maximize their potential in cancer immunotherapy.

## Infected Cell Recognition

Multiple lines of evidence since the late 1980s have shown that γδ T cells display strong activities against bacteria, including *Mycobacterium tuberculosis* (76–81); parasites, such as *Plasmodium falciparum* (82–86); and viruses (87, 88), most notably CMV (89–91).

Vγ9Vδ2 T cells can be specifically and potently activated by PAgs like (E)-4-hydroxy-3-methyl-but-2-enyl pyrophosphate, an intermediate of the 2-C-methyl-d-erythritol 4-phosphate pathway employed by eubacteria and apicomplexan protozoa but not by eukaryotes (48, 92, 93). This likely underlies the striking expansions of Vγ9Vδ2 T cells in individuals infected with *M. tuberculosis* (76–81) or *P. falciparum* (83). Besides PAgs, several other molecules of microbial origin have been proposed as γδ T cell antigens accounting for the specific recognition of infected cells. These candidates include the bacterial superantigens SEA (and to a lesser extent SEE) (19); OXYS and DXS2, two mycobacterial proteins found to activate γδ T cells from BCG-infected human subjects but not from healthy donors (20, 21); and HSV-1 glycoprotein I, specifically recognized by a Vγ1.2Vδ8 TCR independently from antigen processing and MHC presentation (20, 21).

Subsequent reports demonstrated that γδ T cells also recognize stress antigens of cellular origin, either in antibody-like or antigen-presentation-like fashion. γδ T cells can indeed directly recognize stress proteins like hMSH2, a nuclear protein ectopically expressed on the cell surface of different epithelial tumor cells and induced by EBV transformation (22); and Annexin A2 whose expression was induced by CMV infection and recognized specifically by a Vγ8Vδ3 T cell clone (13). On the other hand, γδ T cells can recognize nonpolymorphic MHC-like (class Ib) proteins presenting lipids, such as CD1 proteins, in a similar way to other unconventional T cells like NKT or MAIT cells (11, 94–96). In particular, a subpopulation of Vδ1^+^ T cells has been clearly shown to bind CD1d loaded with the self-lipid sulfatide (97) but any concrete link to the recognition of infected (or transformed) cells remains to be established. Of note, another CD1-like protein, EPCR, was shown to bind directly (independently of lipid cargo) the TCR of a Vγ4Vδ5 T cell clone (expanded from a CMV^+^ individual), thus allowing it to recognize endothelial cells infected with CMV (12).

In addition to the TCR, γδ T cells can also use NKG2D to recognize cells infected with various viruses and intracellular bacteria (32, 98–102). More specifically, the stress-inducible molecule, MICA, was induced on the surface of dendritic and epithelial cells by *M. tuberculosis* infection *in vitro* and *in vivo*; and its binding to NKG2D, substantially enhanced the TCR-dependent Vγ9Vδ2 T cell response to PAgs (28). Furthermore, in the case of *Brucella*, ULBP1 was the main NKG2D ligand upregulated on infected macrophages, and its engagement promoted Vγ9Vδ2 T cell cytotoxicity and cytokine production, which contributed to the inhibition of bacterium development (100).

A few other receptors have implicated in γδ T cell recognition of infected cells. Thus, another NKR, NKG2C, constitutively expressed on Vδ1^+^ T cells, induced a cytolytic response against HIV-infected CD4^+^ T cells expressing its ligand, HLA-E (23). On the other hand, we found that NKp30 can also play an important role in HIV-1 infection upon its induced expression in Vδ1^+^ T cells; NKp30 ligation triggered the production of CCL3, CCL4, and CCL5 chemokines that suppressed the replication of a CCR5 tropic strain of HIV-1 (40). Finally, in the case of avian influenza (H5N1), γδ T cells were reported to use sialic acid receptors for the recognition of viral hemagglutinin (24). To understand how different microorganisms may elicit distinct pathways of γδ T cell recognition of pathogen-associated or stress-induced antigens remains a challenge for future research.

## Concluding Remarks

In contrast with the well-established paradigm of MHC-restricted recognition of peptides by conventional αβ T cells, or even MHC class Ib-dependent recognition of lipids by unconventional αβ T cells, the molecular mechanisms of target cell recognition by γδ T cells remain poorly understood. A notable exception is the BTN3A1-mediated sensing of PAgs by Vγ9Vδ2 T cells, which underlies their responses to tumors and infections like TB or malaria. For most other γδ T cell subsets, however, TCR specificities are either unknown, not generalizable or of unclear physiological relevance. Therefore, the identification of relevant, non-Vγ9Vδ2 TCR ligands remains a major challenge in the γδ T cell field.

On the other hand, while NKRs are also clearly involved in γδ T cell recognition of tumor or infected cells, we still lack appropriate understanding how the multiple signals derived from all the expressed NKRs are integrated, also with those coming from the TCR itself. This likely depends on the relative expression levels of the various putative NKR and TCR ligands in each target cell, which adds significant complexity to the process of γδ T cell recognition.

The broad spectrum of MHC-unrestricted recognition of infected or transformed cells by γδ T makes them attractive candidates for adoptive cell therapy (ACT). All clinical trials have thus far concentrated on Vγ9Vδ2 T cells, probably due to their relative abundance in the peripheral blood and especially the availability of FDA-approved drugs, such as zoledronate and pamidronate, that allow their activation and expansion *in vivo* (103). Vγ9Vδ2 ACT has shown promising pre-clinical results against TB (104) and has already been tested in various cancer clinical trials [reviewed in Ref. (105)] that documented its safety and some (albeit still sub-optimal) efficacy (106–108). This could be maybe explained by Vγ9Vδ2 T cell susceptibility to exhaustion and activation-induced cell death (AICD). Nonetheless, improvements in Vγ9Vδ2 ACT protocols may still increase their efficacy, as indicated by some studies with exogenous provision of IL-2, importantly without the need for lymphodepleting preconditioning (109, 110). As for Vδ1^+^ γδ T cells, they are less susceptible to AICD and exhaustion when compared to Vγ9Vδ2 T cells (111). However, no clinical trial has yet focused on this γδ T cell subset, mostly due to the lack of clinical-grade protocols allowing their successful expansion. Importantly, we have recently developed a clinical-grade process to effectively expand Vδ1^+^ T cells while also inducing NCR (and augmenting NKG2D) expression; and established the proof-of-concept in leukemia xenograft models (73). We further anticipate NCR^+^ Vδ1^+^ ACT to be a promising therapeutic strategy also for solid tumors and chronic viral infections.

## Author Contributions

AS, BL, and BS-S conceived and wrote the manuscript. AS and BL contributed equally to the manuscript.

## Conflict of Interest Statement

BS-S is a co-founder and shareholder of Lymphact—Lymphocyte Activation Technologies S.A. The other authors declare that the research was conducted in the absence of any commercial or financial relationships that could be construed as a potential conflict of interest.

## References

[1] Pardoll DM Fowlkes BJ Bluestone JA Kruisbeek A Maloy WL Coligan JE Differential expression of two distinct T-cell receptors during thymocyte development. Nature (1987) 326:79–82. 10.1038/326079a0 3102971

[2] De Rosa SC Andrus JP Perfetto SP Mantovani JJ Herzenberg LA Roederer M Ontogeny of T cells in humans. J Immunol (2004) 172:1637–45. 10.4049/jimmunol.172.3.1637 14734745

[3] Ribeiro ST Ribot JC Silva-Santos B Five layers of receptor signaling in γδ T-cell differentiation and activation. Front Immunol (2015) 6:15 10.3389/fimmu.2015.00015 25674089 PMC4306313

[4] Carding SR Egan PJ γδ T cells: functional plasticity and heterogeneity. Nat Rev Immunol (2002) 2:336–45. 10.1038/nri797 12033739

[5] Karunakaran MM Herrmann T. The Vγ9Vδ2 T cell antigen receptor and butyrophilin-3 A1: models of interaction, the possibility of co-evolution, and the case of dendritic epidermal T cells. Front Immunol (2014) 5:648. 10.3389/fimmu.2014.00648 25566259 PMC4271611

[6] Khairallah C Déchanet-Merville J Capone M γδ T cell-mediated immunity to cytomegalovirus infection. Front Immunol (2017) 8:105 10.3389/fimmu.2017.00105 28232834 PMC5298998

[7] Ravens S Schultze-Florey C Raha S Sandrock I Drenker M Oberdörfer L Human γδ T cells are quickly reconstituted after stem-cell transplantation and show adaptive clonal expansion in response to viral infection. Nat Immunol (2017) 18:393–401. 10.1038/ni.3686 28218745

[8] Gu S Nawrocka W Adams EJ Sensing of pyrophosphate metabolites by Vγ9Vδ2 T cells. Front Immunol (2015) 5:688 10.3389/fimmu.2014.00688 25657647 PMC4303140

[9] Hayday AC γδ T cells and the lymphoid stress-surveillance response. Immunity (2009) 31:184–96. 10.1016/j.immuni.2009.08.006 19699170

[10] Sieling PA Jullien D Dahlem M Tedder TF Rea TH Modlin RL CD1 expression by dendritic cells in human leprosy lesions: correlation with effective host immunity. J Immunol (1999) 162:1851–8. 9973451

[11] Russano AM Bassotti G Agea E Bistoni O Mazzocchi A Morelli A CD1-restricted recognition of exogenous and self-lipid antigens by duodenal γδ+ T lymphocytes. J Immunol (2007) 178:3620–6. 10.4049/jimmunol.178.6.3620 17339459

[12] Willcox CR Pitard V Netzer S Couzi L Salim M Silberzahn T Cytomegalovirus and tumor stress surveillance by binding of a human γδ T cell antigen receptor to endothelial protein C receptor. Nat Immunol (2012) 13:872–9. 10.1038/ni.2394 22885985

[13] Marlin R Pappalardo A Kaminski H Willcox CR Pitard V Netzer S Sensing of cell stress by human γδ TCR-dependent recognition of annexin A2. Proc Natl Acad Sci U S A (2017) 114:3163–8. 10.1073/pnas.1621052114 28270598 PMC5373368

[14] Chen H He X Wang Z Wu D Zhang H Xu C Identification of human T cell receptor γδ-recognized epitopes/proteins via CDR3δ peptide-based immunobiochemical strategy. J Biol Chem (2008) 283:12528–37. 10.1074/jbc.M708067200 18321859

[15] Laad AD Thomas ML Fakih AR Chiplunkar SV. Human gamma delta T cells recognize heat shock protein-60 on oral tumor cells. Int J Cancer (1999) 80:709–14. 10.1002/(SICI)1097-0215(19990301)80:5<709::AID-IJC14>3.0.CO;2-R 10048972

[16] Kaur I Voss SD Gupta RS Schell K Fisch P Sondel PM. Human peripheral gamma delta T cells recognize hsp60 molecules on Daudi Burkitt’s lymphoma cells. J Immunol (1993) 150:2046–55. 8094731

[17] Fisch P Malkovsky M Kovats S Sturm E Braakman E Klein BS Recognition by human V gamma 9/V delta 2 T cells of a GroEL homolog on Daudi Burkitt’s lymphoma cells. Science (1990) 250:1269–73. 10.1126/science.1978758 1978758

[18] Scotet E Martinez LO Grant E Barbaras R Jenö P Guiraud M Tumor recognition following Vγ9Vδ2 T cell receptor interactions with a surface F1-ATPase-related structure and apolipoprotein A-I. Immunity (2005) 22:71–80. 10.1016/j.immuni.2004.11.012 15664160

[19] Rust C Koning F γδ T cell reactivity towards bacterial superantigens. Semin Immunol (1993) 5:41–6. 10.1006/smim.1993.1006 8467094

[20] Xi XY Zhang XY Wang B Wang J Huang H Cui LX A novel strategy to screen bacillus Calmette-Guérin protein antigen recognized by γδ TCR. PLoS One (2011) 6:e18809 10.1371/journal.pone.0018809 21526117 PMC3081299

[21] Xi X Han X Li L Zhao Z Identification of a new tuberculosis antigen recognized by γδ T cell receptor. Clin Vaccine Immunol (2013) 20:530–9. 10.1128/CVI.00584-12 23389928 PMC3623398

[22] Dai Y Chen H Mo C Cui L He W Ectopically expressed human tumor biomarker MutS homologue 2 is a novel endogenous ligand that is recognized by human γδ T cells to induce innate anti-tumor/virus immunity. J Biol Chem (2012) 287:16812–9. 10.1074/jbc.M111.327650 22433851 PMC3351303

[23] Fausther-Bovendo H Wauquier N Cherfils-Vicini J Cremer I Debré P Vieillard V NKG2C is a major triggering receptor involved in the Vδ1 T cell-mediated cytotoxicity against HIV-infected CD4 T cells. AIDS (2008) 22:217–26. 10.1097/QAD.0b013e3282f46e7c 18097224

[24] Lu Y Li Z Ma C Wang H Zheng J Cui L The interaction of influenza H5N1 viral hemagglutinin with sialic acid receptors leads to the activation of human γδ T cells. Cell Mol Immunol (2013) 10:463–70. 10.1038/cmi.2013.26 23912782 PMC4002388

[25] Mami-Chouaib F Miossec C Del Porto P Flament C Triebel F Hercend T. T cell target 1 (TCT.1): a novel target molecule for human non-major histocompatibility complex-restricted T lymphocytes. J Exp Med (1990) 172:1071–82. 10.1084/jem.172.4.1071 2212943 PMC2188610

[26] Mami-Chouaib F Del Porto P Delorme D Hercend T. Further evidence for a gamma/delta T cell receptor-mediated TCT.1/CD48 recognition. J Immunol (1991) 147:2864–7. 1655899

[27] Flament C Bellagha K Rosenthal-Allieri A Chouaib S Mami-Chouaib F CD48 may serve as an accessory molecule for the activation of a subset of human γ/δ T cells. Hum Immunol (1996) 46:82–92. 10.1016/0198-8859(96)00010-9 8727206

[28] Groh V Steinle A Bauer S Spies T Recognition of stress-induced MHC molecules by intestinal epithelial γδ T cells. Science (1998) 279:1737–40. 10.1126/science.279.5357.1737 9497295

[29] Wu J Groh V Spies T. T cell antigen receptor engagement and specificity in the recognition of stress-inducible MHC class I-related chains by human epithelial gamma delta T cells. J Immunol (2002) 169:1236–40. 10.4049/jimmunol.169.3.1236 12133944

[30] Xu B Pizarro JC Holmes MA McBeth C Groh V Spies T Crystal structure of a T-cell receptor specific for the human MHC class I homolog MICA. Proc Natl Acad Sci U S A (2011) 108:2414–9. 10.1073/pnas.1015433108 21262824 PMC3038733

[31] Bauer S Groh V Wu J Steinle A Phillips JH Lanier LL Activation of NK cells and T cells by NKG2D, a receptor for stress-inducible MICA. Science (1999) 285:727–9. 10.1126/science.285.5428.727 10426993

[32] Das H Groh V Kuijl C Sugita M Morita CT Spies T MICA engagement by human Vgamma2Vdelta2 T cells enhances their antigen-dependent effector function. Immunity (2001) 15:83–93. 10.1016/S1074-7613(01)00168-6 11485740

[33] Lu J Das M Kanji S Aggarwal R Joseph M Ray A Induction of ATM/ATR pathway combined with Vγ2Vδ2 T cells enhances cytotoxicity of ovarian cancer cells. Biochim Biophys Acta (2014) 1842:1071–9. 10.1016/j.bbadis.2014.04.003 24726882 PMC4082796

[34] Lança T Correia DV Moita CF Raquel H Neves-Costa A Ferreira C The MHC class Ib protein ULBP1 is a nonredundant determinant of leukemia/lymphoma susceptibility to γδ T-cell cytotoxicity. Blood (2010) 115:2407–11. 10.1182/blood-2009-08-237123 20101024

[35] Catellani S Poggi A Bruzzone A Dadati P Ravetti JL Gobbi M Expansion of Vδ1 T lymphocytes producing IL-4 in low-grade non-Hodgkin lymphomas expressing UL-16-binding proteins. Blood (2007) 109:2078–85. 10.1182/blood-2006-06-028985 16973957

[36] Bryant NL Gillespie GY Lopez RD Markert JM Cloud GA Langford CP Preclinical evaluation of ex vivo expanded/activated gammadelta T cells for immunotherapy of glioblastoma multiforme. J Neurooncol (2011) 101:179–88. 10.1007/s11060-010-0245-2 20532954 PMC13015848

[37] Poggi A Venturino C Catellani S Clavio M Miglino M Gobbi M Vδ1 T lymphocytes from B-CLL patients recognize ULBP3 expressed on leukemic B cells and up-regulated by trans-retinoic acid. Cancer Res (2004) 64:9172–9. 10.1158/0008-5472.CAN-04-2417 15604289

[38] Kong Y Cao W Xi X Ma C Cui L He W The NKG2D ligand ULBP4 binds to TCRγ9/δ2 and induces cytotoxicity to tumor cells through both TCRγδ and NKG2D. Blood (2009) 114:310–7. 10.1182/blood-2008-12-196287 19436053

[39] Correia DV Fogli M Hudspeth K Gomes Da Silva M Mavilio D Silva-Santos B Differentiation of human peripheral blood Vδ1+ T cells expressing the natural cytotoxicity receptor NKp30 for recognition of lymphoid leukemia cells. Blood (2011) 118:992–1001. 10.1182/blood-2011-02-339135 21633088

[40] Hudspeth K Fogli M Correia DV Mikulak J Roberto A Della S Engagement of NKp30 on Vδ1 T-cells induces the production of CCL3, CCL4 and CCL5 and suppresses HIV-1 replication. Blood (2012) 119:4013–6. 10.1182/blood-2011-11-390153 22403253

[41] Toutirais O Cabillic F Le Friec G Salot S Loyer P Le Gallo M DNAX accessory molecule-1 (CD226) promotes human hepatocellular carcinoma cell lysis by Vγ9Vδ2 T cells. Eur J Immunol (2009) 39:1361–8. 10.1002/eji.200838409 19404979

[42] Sturm E Braakman E Fisch P Vreugdenhil RJ Sondel P Bolhuis RL. Human V gamma 9-V delta 2 T cell receptor-gamma delta lymphocytes show specificity to Daudi Burkitt’s lymphoma cells. J Immunol (1990) 145:3202–8. 2146317

[43] Fisch P Oettel K Fudim N Surfus JE Malkovsky M Sondel PM. MHC-unrestricted cytotoxic and proliferative responses of two distinct human gamma/delta T cell subsets to Daudi cells. J Immunol (1992) 148:2315–23. 1532810

[44] Weintraub BC Jackson MR Hedrick SM. Gamma delta T cells can recognize nonclassical MHC in the absence of conventional antigenic peptides. J Immunol (1994) 153:3051–8. 8089486

[45] Tanaka Y Morita CT Tanaka Y Nieves E Brenner MB Bloom BR. Natural and synthetic non-peptide antigens recognized by human gamma delta T cells. Nature (1995) 375:155–8. 10.1038/375155a0 7753173

[46] Morita CT Beckman EM Bukowski JF Tanaka Y Band H Bloom BR Direct presentation of nonpeptide prenyl pyrophosphate antigens to human γδ T cells. Immunity (1995) 3:495–507. 10.1016/1074-7613(95)90178-7 7584140

[47] Jomaa H Feurle J Lühs K Kunzmann V Tony HP Herderich M Vγ9/Vδ2 T cell activation induced by bacterial low molecular mass compounds depends on the 1-deoxy-D-xylulose 5-phosphate pathway of isoprenoid biosynthesis. FEMS Immunol Med Microbiol (1999) 25:371–8. 10.1016/S0928-8244(99)00110-8 10497868

[48] Thedrez A Sabourin C Gertner J Devilder MC Allain-Maillet S Fournie JJ Self/non-self discrimination by human gammadelta T cells: simple solutions for a complex issue? Immunol Rev (2007) 215:123–35. 10.1111/j.1600-065X.2006.00468.x 17291284

[49] Gober H-J Kistowska M Angman L Jenö P Mori L De Libero G. Human T cell receptor gammadelta cells recognize endogenous mevalonate metabolites in tumor cells. J Exp Med (2003) 197:163–8. 10.1084/jem.20021500 12538656 PMC2193814

[50] Todaro M D’Asaro M Caccamo N Iovino F Francipane MG Meraviglia S Efficient killing of human colon cancer stem cells by gammadelta T lymphocytes. J Immunol (2009) 182:7287–96. 10.4049/jimmunol.0804288 19454726

[51] Vavassori S Kumar A Wan GS Ramanjaneyulu GS Cavallari M El Daker S Butyrophilin 3A1 binds phosphorylated antigens and stimulates human γδ T cells. Nat Immunol (2013) 14:908–16. 10.1038/ni.2665 23872678

[52] Harly C Guillaume Y Nedellec S Peigné CM Mönkkönen H Mönkkönen J Key implication of CD277/butyrophilin-3 (BTN3A) in cellular stress sensing by a major human γδ T-cell subset. Blood (2012) 120:2269–79. 10.1182/blood-2012-05-430470 22767497 PMC3679641

[53] Sandstrom A Peigné CM Léger A Crooks J Konczak F Gesnel MC The intracellular B30.2 domain of butyrophilin 3A1 binds phosphoantigens to mediate activation of human Vγ9Vδ2T cells. Immunity (2014) 40:490–500. 10.1016/j.immuni.2014.03.003 24703779 PMC4028361

[54] Peigné C-M Léger A Gesnel M-C Konczak F Olive D Bonneville M The juxtamembrane domain of butyrophilin BTN3A1 controls phosphoantigen-mediated activation of human Vγ9Vδ2 T cells. J Immunol (2017) 198:4228–34. 10.4049/jimmunol.1601910 28461569

[55] Gu S Sachleben JR Boughter CT Nawrocka WI Borowska MT Tarrasch JT Phosphoantigen-induced conformational change of butyrophilin 3A1 (BTN3A1) and its implication on Vγ9Vδ2 T cell activation. Proc Natl Acad Sci U S A (2017) 114:E7311–20. 10.1073/pnas.1707547114 28807997 PMC5584448

[56] Sebestyen Z Scheper W Vyborova A Gu S Rychnavska Z Schiffler M RhoB mediates phosphoantigen recognition by Vγ9Vδ2 T cell receptor. Cell Rep (2016) 15:1973–85. 10.1016/j.celrep.2016.04.081 27210746 PMC5035041

[57] Garni-Wagner BA Purohit A Mathew PA Bennett M Kumar V. A novel function-associated molecule related to non-MHC-restricted cytotoxicity mediated by activated natural killer cells and T cells. J Immunol (1993) 151:60–70. 8326140

[58] Schuhmachers G Ariizumi K Mathew PA Bennett M Kumar Y Takashima A Activation of murine epidermal γδ T cells through surface 2B4. Eur J Immunol (1995) 25:1117–20. 10.1002/eji.1830250440 7737283

[59] Nakajima H Cella M Langen H Friedlein A Colonna M. Activating interactions in human NK cell recognition: the role of 2B4-CD48. Eur J Immunol (1999) 29:1676–83. 10.1002/(SICI)1521-4141(199905)29:05<1676::AID-IMMU1676>3.0.CO;2-Y 10359122

[60] Steinle A Li P Morris DL Groh V Lanier LL Strong RK Interactions of human NKG2D with its ligands MICA, MICB, and homologs of the mouse RAE-1 protein family. Immunogenetics (2001) 53:279–87. 10.1007/s002510100325 11491531

[61] Von Lilienfeld-Toal M Nattermann J Feldmann G Sievers E Frank S Strehl J Activated γδ T cells express the natural cytotoxicity receptor natural killer p44 and show cytotoxic activity against myeloma cells. Clin Exp Immunol (2006) 144:528–33. 10.1111/j.1365-2249.2006.03078.x 16734623 PMC1941970

[62] Groh V Rhinehart R Secrist H Bauer S Grabstein KH Spies T. Broad tumor-associated expression and recognition by tumor-derived gamma delta T cells of MICA and MICB. Proc Natl Acad Sci U S A (1999) 96:6879–84. 10.1073/pnas.96.12.6879 10359807 PMC22010

[63] Wrobel P Shojaei H Schittek B Gieseler F Wollenberg B Kalthoff H Lysis of a broad range of epithelial tumour cells by human γδ T cells: involvement of NKG2D ligands and T-cell receptor- versus NKG2D-dependent recognition. Scand J Immunol (2007) 66:320–8. 10.1111/j.1365-3083.2007.01963.x 17635809

[64] Deniger DC Maiti SN Mi T Switzer KC Ramachandran V Hurton LV Activating and propagating polyclonal gamma delta T cells with broad specificity for malignancies. Clin Cancer Res (2014) 20:5708–19. 10.1158/1078-0432.CCR-13-3451 24833662 PMC4233015

[65] Gertner-dardenne J Castellano R Mamessier E Garbit S Kochbati E Etienne A Human Vγ9Vδ2 T cells specifically recognize and kill acute myeloid leukemic blasts. J Immunol (2012) 188:4701–8. 10.4049/jimmunol.1103710 22467661

[66] Knight A MacKinnon S Lowdell MW. Human Vdelta1 gamma-delta T cells exert potent specific cytotoxicity against primary multiple myeloma cells. Cytotherapy (2012) 14:1110–8. 10.3109/14653249.2012.700766 22800570

[67] Chitadze G Lettau M Luecke S Wang T Janssen O Fürst D NKG2D- and T-cell receptor-dependent lysis of malignant glioma cell lines by human γδ T cells: modulation by temozolomide and A disintegrin and metalloproteases 10 and 17 inhibitors. Oncoimmunology (2016) 5:e1093276 10.1080/2162402X.2015.1093276 27141377 PMC4839372

[68] Niu C Jin H Li M Zhu S Zhou L Jin F Low-dose bortezomib increases the expression of NKG2D and DNAM-1 ligands and enhances induced NK and γδ T cell-mediated lysis in multiple myeloma. Oncotarget (2016) 8:5954–64. 10.18632/oncotarget.13979 PMC5351604 27992381

[69] Peipp M Wesch D Oberg HH Lutz S Muskulus A van de Winkel JGJ CD20-specific immunoligands engaging NKG2D enhance γδ T cell-mediated lysis of lymphoma cells. Scand J Immunol (2017) 86:196–206. 10.1111/sji.12581 28708284

[70] Todaro M Orlando V Cicero G Caccamo N Meraviglia S Stassi G Chemotherapy sensitizes colon cancer initiating cells to Vγ9Vδ2 T cell-mediated cytotoxicity. PLoS One (2013) 8:e65145 10.1371/journal.pone.0065145 23762301 PMC3675136

[71] Hudspeth K Silva-Santos B Mavilio D. Natural cytotoxicity receptors: broader expression patterns and functions in innate and adaptive immune cells. Front Immunol (2013) 4:69. 10.3389/fimmu.2013.00069 23518691 PMC3603285

[72] Cantoni C Bottino C Vitale M Pessino A Augugliaro R Malaspina A NKp44, a triggering receptor involved in tumor cell lysis by activated human natural killer cells, is a novel member of the immunoglobulin superfamily. J Exp Med (1999) 189:787–95. 10.1084/jem.189.5.787 10049942 PMC2192947

[73] Almeida AR Correia DV Fernandes-Platzgummer A Da Silva CL Da Silva MG Anjos DR Delta one T cells for immunotherapy of chronic lymphocytic leukemia: clinical-grade expansion/differentiation and preclinical proof of concept. Clin Cancer Res (2016) 22:5795–804. 10.1158/1078-0432.CCR-16-0597 27307596

[74] Brandt CS Baratin M Yi EC Kennedy J Gao Z Fox B The B7 family member B7-H6 is a tumor cell ligand for the activating natural killer cell receptor NKp30 in humans. J Exp Med (2009) 206:1495–503. 10.1084/jem.20090681 19528259 PMC2715080

[75] Barrow AD Edeling MA Trifonov V Luo J Goyal P Bohl B Natural killer cells control tumor growth by sensing a growth factor. Cell (2018) 172:534–48.e19. 10.1016/j.cell.2017.11.037 29275861 PMC6684025

[76] Young JL Goodall JC Beacock-Sharp H Gaston JS. Human gamma delta T-cell recognition of *Yersinia enterocolitica*. Immunology (1997) 91:503–10. 10.1046/j.1365-2567.1997.00289.x 9378487 PMC1363868

[77] Barisa M Kramer AM Majani Y Moulding D Saraiva L Bajaj-Elliott M *E. coli* promotes human Vγ9Vδ2 T cell transition from cytokine-producing bactericidal effectors to professional phagocytic killers in a TCR-dependent manner. Sci Rep (2017) 7:1–12. 10.1038/s41598-017-02886-8 28584241 PMC5459831

[78] Hara T Mizuno Y Takaki K Takada H Akeda H Aoki T Predominant activation and expansion of V gamma 9-bearing gamma delta T cells in vivo as well as in vitro in *Salmonella* infection. J Clin Invest (1992) 90:204–10. 10.1172/JCI115837 1386086 PMC443082

[79] Munk ME Elser C Kaufmann SHE Human y/δ T-cell response to *Listeria monocytogenes* protein components in vitro. Immunology (1996) 230:230–5. 10.1046/j.1365-2567.1996.470549.x PMC1384278 8698384

[80] Marx S Wesch D Kabelitz D. Activation of human gamma delta T cells by *Mycobacterium tuberculosis* and Daudi lymphoma cells: differential regulatory effect of IL-10 and IL-12. J Immunol (1997) 158:2842–8. 9058820

[81] Chen ZW. Protective immune responses of major Vγ2Vδ2 T-cell subset in *M. tuberculosis* infection. Curr Opin Immunol (2016) 42:105–12. 10.1016/j.coi.2016.06.005 27491008 PMC5754217

[82] Hara T Ohashi S Yamashita Y Abe T Hisaeda H Himeno K Human V delta 2+ gamma delta T-cell tolerance to foreign antigens of *Toxoplasma gondii*. Proc Natl Acad Sci U S A (1996) 93:5136–40. 10.1073/pnas.93.10.5136 8643541 PMC39420

[83] Kurup SP Harty JT γδ T cells and immunity to human malaria in endemic regions. Ann Transl Med (2015) 3:S22 10.3978/j.issn.2305-5839.2015.02.22 26046068 PMC4437949

[84] Russo DM Armitage RJ Barral-Netto M Barral A Grabstein KH Reed SG. Antigen-reactive gamma delta T cells in human leishmaniasis. J Immunol (1993) 151:3712–8. 8376802

[85] Elloso MM van der Heyde HC vande Waa JA Manning DD Weidanz WP. Inhibition of *Plasmodium falciparum* in vitro by human gamma delta T cells. J Immunol (1994) 153:1187–94. 8027548

[86] Giulia C Loizon S Guenot M Mocan I Halary F De Saint-Basile G Control of *Plasmodium falciparum* erythrocytic cycle: γδ T cells target the red blood cell-invasive merozoites. Blood (2011) 118:6952–62. 10.1182/blood-2011-08-376111 22045985

[87] Poccia F Agrati C Martini F Capobianchi MR Wallace M Malkovsky M Antiviral reactivities of γδ T cells. Microbes Infect (2005) 7:518–28. 10.1016/j.micinf.2004.12.009 15777667 PMC7110461

[88] Cimini E Viola D Cabeza-Cabrerizo M Romanelli A Tumino N Sacchi A Different features of Vδ2 T and NK cells in fatal and non-fatal human Ebola infections. PLoS Negl Trop Dis (2017) 11:e0005645. 10.1371/journal.pntd.0005645 28558022 PMC5472323

[89] Knight A Madrigal AJ Grace S Sivakumaran J Kottaridis P Mackinnon S The role of Vδ2-negative γδ T cells during cytomegalovirus reactivation in recipients of allogeneic stem cell transplantation. Blood (2010) 116:2164–72. 10.1182/blood-2010-01-255166 20576814

[90] Pitard V Roumanes D Lafarge X Couzi L Garrigue I Lafon ME Long-term expansion of effector/memory V{delta}2-{gamma}-{delta} T cells is a specific blood signature of CMV infection. Blood (2008) 112:1317–24. 10.1182/blood-2008-01-136713 18539896 PMC2515135

[91] Lafarge X Merville P Cazin MC Bergé F Potaux L Moreau JF Cytomegalovirus infection in transplant recipients resolves when circulating gammadelta T lymphocytes expand, suggesting a protective antiviral role. J Infect Dis (2001) 184:533–41. 10.1086/322843 11494158

[92] Eberl M Hintz M Reichenberg A Kollas AK Wiesner J Jomaa H Microbial isoprenoid biosynthesis and human γδ T cell activation. FEBS Lett (2003) 544:4–10. 10.1016/S0014-5793(03)00483-6 12782281

[93] Hintz M Reichenberg A Altincicek B Bahr U Gschwind RM Kollas AK Identification of (E)-4-hydroxy-3-methyl-but-2-enyl pyrophosphate as a major activator for human γδ T cells in *Escherichia coli*. FEBS Lett (2001) 509:317–22. 10.1016/S0014-5793(01)03191-X 11741609

[94] Gao Y Williams AP. Role of innate T cells in anti-bacterial immunity. Front Immunol (2015) 6:302. 10.3389/fimmu.2015.00302 26124758 PMC4463001

[95] Pellicci DG Uldrich AP Le Nours J Ross F Chabrol E Eckle SB The molecular bases of delta/alphabeta T cell-mediated antigen recognition. J Exp Med (2014) 211:2599–615. 10.1084/jem.20141764 25452463 PMC4267242

[96] Uldrich AP Le Nours J Pellicci DG Gherardin NA Mcpherson KG Lim RT CD1d-lipid antigen recognition by the γδ TCR. Nat Immunol (2013) 14:1137–45. 10.1038/ni.2713 24076636

[97] Luoma AM Castro CD Mayassi T Bembinster LA Bai L Picard D Crystal structure of Vδ1 T cell receptor in complex with CD1d-sulfatide shows MHC-like recognition of a self-lipid by human γδ T cells. Immunity (2013) 39:1032–42. 10.1016/j.immuni.2013.11.001 24239091 PMC3875342

[98] Agrati C D’Offizi G Narciso P Abrignani S Ippolito G Colizzi V Vdelta1 T lymphocytes expressing a Th1 phenotype are the major gammadelta T cell subset infiltrating the liver of HCV-infected persons. Mol Med (2001) 7:11–9. 11474123 PMC1949990

[99] Li H Xiang Z Feng T Li J Liu Y Fan Y Human Vγ9Vδ2-T cells efficiently kill influenza virus-infected lung alveolar epithelial cells. Cell Mol Immunol (2013) 10:159–64. 10.1038/cmi.2012.70 23353835 PMC4003054

[100] Bessoles S Ni M Garcia-Jimenez S Sanchez F Lafont V. Role of NKG2D and its ligands in the anti-infectious activity of Vγ9Vδ2 T cells against intracellular bacteria. Eur J Immunol (2011) 41:1619–28. 10.1002/eji.201041230 21469127

[101] Bieback K Breer C Nanan R Ter Meulen V Schneider-Schaulies S. Expansion of human gamma/delta T cells in vitro is differentially regulated by the measles virus glycoproteins. J Gen Virol (2003) 84:1179–88. 10.1099/vir.0.19027-0 12692283

[102] Knight A Arnouk H Britt W Gillespie GY Cloud GA Harkins L CMV-independent lysis of glioblastoma by ex vivo expanded/activated Vδ1+ γδ T cells. PLoS One (2013) 8:e68729. 10.1371/journal.pone.0068729 23950874 PMC3737218

[103] Kunzmann V Bauer E Wilhelm M γ/δ T-cell stimulation by pamidronate. N Engl J Med (1999) 340:737–8. 10.1056/NEJM199903043400914 10068336

[104] Qaqish A Huang D Chen CY Zhang Z Wang R Li S Adoptive transfer of phosphoantigen-specific γδ T cell subset attenuates mycobacterium tuberculosis infection in nonhuman primates. J Immunol (2017) 198:4753–63. 10.4049/jimmunol.1602019 28526681 PMC5557270

[105] Fisher JP Heuijerjans J Yan M Gustafsson K Anderson J γδ T cells for cancer immunotherapy. Oncoimmunology (2014) 3:e27572 10.4161/onci.27572 24734216 PMC3984269

[106] Fournié JJ Sicard H Poupot M Bezombes C Blanc A Romagné F What lessons can be learned from γδ T cell-based cancer immunotherapy trials? Cell Mol Immunol (2013) 10:35–41. 10.1038/cmi.2012.39 23241899 PMC4003170

[107] Legut M Cole DK Sewell AK The promise of γδT cells and the γδT cell receptor for cancer immunotherapy. Cell Mol Immunol (2015) 12:656–8. 10.1038/cmi.2015.28 25864915 PMC4716630

[108] Zou C Zhao P Xiao Z Han X Fu F Fu L γδ T cells in cancer immunotherapy. Oncotarget (2017) 8:8900–9. 10.18632/oncotarget.13051 27823972 PMC5352452

[109] Nakajima J Murakawa T Fukami T Goto S Kaneko T Yoshida Y A phase I study of adoptive immunotherapy for recurrent non-small-cell lung cancer patients with autologous γδ T cells. Eur J Cardiothorac Surg (2010) 37:1191–7. 10.1016/j.ejcts.2009.11.051 20137969

[110] Izumi T Kondo M Takahashi T Fujieda N Kondo A Tamura N Ex vivo characterization of γδ T-cell repertoire in patients after adoptive transfer of Vγ9Vδ2 T cells expressing the interleukin-2 receptor β-chain and the common γ-chain. Cytotherapy (2013) 15:481–91. 10.1016/j.jcyt.2012.12.004 23391461

[111] Siegers GM Lamb LS Cytotoxic and regulatory properties of circulating Vδ1+ γδ t cells: a new player on the cell therapy field? Mol Ther (2014) 22:1416–22. 10.1038/mt.2014.104 24895997 PMC4435582

